# Using machine learning-based algorithms to construct cardiovascular risk prediction models for Taiwanese adults based on traditional and novel risk factors

**DOI:** 10.1186/s12911-024-02603-2

**Published:** 2024-07-22

**Authors:** Chien-Hsiang Cheng, Bor-Jen Lee, Oswald Ndi Nfor, Chih-Hsuan Hsiao, Yi-Chia Huang, Yung-Po Liaw

**Affiliations:** 1 Department of Respiratory Therapy, Taichung Veterans General Hospital, Taichung, 40705 Taiwan; 2 Department of Critical Care Medicine, Tungs’ Taichung Metroharbor Hospital, Taichung, Taiwan; 3 Department of Public Health, Institute of Public Health, Chung Shan Medical University, No. 110, Sec. 1 Jianguo N. Rd, Taichung City, 40201 Taiwan; 4 Department of Nutrition, Chung Shan Medical University and Chung Shan Medical University Hospital, No. 110, Sec. 1 Jianguo N. Rd, Taichung, 40201 Taiwan; 5 Department of Medical Imaging, Chung Shan Medical University Hospital, Taichung , 40201 Taiwan; 6 Institute of Medicine, Chung Shan Medical University, Taichung, 40201 Taiwan

**Keywords:** Coronary artery disease, Machine learning, Gradient boosting, Taiwan, Risk prediction, Age, Sensitivity, Specificity

## Abstract

**Objective:**

To develop and validate machine learning models for predicting coronary artery disease (CAD) within a Taiwanese cohort, with an emphasis on identifying significant predictors and comparing the performance of various models.

**Methods:**

This study involved a comprehensive analysis of clinical, demographic, and laboratory data from 8,495 subjects in Taiwan Biobank (TWB) after propensity score matching to address potential confounding factors. Key variables included age, gender, lipid profiles (T-CHO, HDL_C, LDL_C, TG), smoking and alcohol consumption habits, and renal and liver function markers. The performance of multiple machine learning models was evaluated.

**Results:**

The cohort comprised 1,699 individuals with CAD identified through self-reported questionnaires. Significant differences were observed between CAD and non-CAD individuals regarding demographics and clinical features. Notably, the Gradient Boosting model emerged as the most accurate, achieving an AUC of 0.846 (95% confidence interval [CI] 0.819–0.873), sensitivity of 0.776 (95% CI, 0.732–0.820), and specificity of 0.759 (95% CI, 0.736–0.782), respectively. The accuracy was 0.762 (95% CI, 0.742–0.782). Age was identified as the most influential predictor of CAD risk within the studied dataset.

**Conclusion:**

The Gradient Boosting machine learning model demonstrated superior performance in predicting CAD within the Taiwanese cohort, with age being a critical predictor. These findings underscore the potential of machine learning models in enhancing the prediction accuracy of CAD, thereby supporting early detection and targeted intervention strategies.

**Trial registration:**

Not applicable.

## Background

The emergence of machine learning (ML) technologies in the medical sector has revolutionized how diseases, particularly CAD, are predicted and managed. CAD has emerged as a primary contributor to the global burden of disease, claiming a significant number of lives annually [1]. In Taiwan, it ranks as the second most common cause of mortality across genders, as reported by the Health Promotion Administration of the Ministry of Health and Welfare in 2020. The most effective approach to mitigate or slow the progression of this disease involves the creation of a robust screening mechanism that can detect cardiovascular risk factors early on.

A plethora of factors including age, gender, obesity, elevated blood pressure levels, dyslipidemia, and glucose anomalies, along with smoking and alcohol consumption behaviors, have been universally recognized as contributors to the risk of developing CAD [2]. The pioneering Framingham Heart Study introduced a cardiovascular risk prediction model, known as the Framingham risk score, utilizing conventional risk indicators (e.g., age, gender, smoking status, HDL cholesterol levels, systolic blood pressure, treatment for hypertension, and diabetes presence) to predict the likelihood of coronary heart disease events, both fatal and non-fatal [3]. It has been previously suggested that the Framingham score encompasses a limited number of predictors and may overestimate CVD risk, potentially leading to overtreatment [4, 5]. Subsequently, several risk prediction models incorporating the aforementioned conventional factors have been formulated to pinpoint individuals at elevated risk for heart diseases [6–11]. While these models offer satisfactory risk predictions with C statistics ranging between 0.65 and 0.85 [12, 13], their derivation from populations of European or American descent raises concerns about their applicability to Asian demographics, potentially leading to inaccurate risk assessments [14–17].

The limitations inherent in these conventional cardiovascular risk prediction models, coupled with the potential for population-specific discrepancies, have been acknowledged [12, 18]. As a result, there has been interest in incorporating novel cardiovascular risk indicators (such as coronary artery calcium scores, carotid intima-media thickness, ankle-brachial index, and flow-mediated dilation) to improve the predictive accuracy of these algorithms [18]. Despite this, enhancements brought about by these novel markers have been marginal or not cost-effective.

In the face of these challenges, the deployment of artificial intelligence (AI) in healthcare, particularly in enhancing the precision of disease prediction, has seen a rapid increase [19–21]. Nonetheless, the particularities of CAD risk factors within the Taiwanese population have not been extensively studied. The application of AI-driven models in cardiovascular disease prediction promises to offer more nuanced risk assessments. This study aims to leverage an extensive set of predictive factors through AI algorithms, thereby enhancing risk stratification and making significant contributions towards the advancement of precision medicine. The goal herein is to discern the attributes associated with CAD and to formulate a risk prediction model tailored to the Taiwanese cohort.

## Materials and methods

### Study population, data source, and outcome variable

**Fig. 1:**
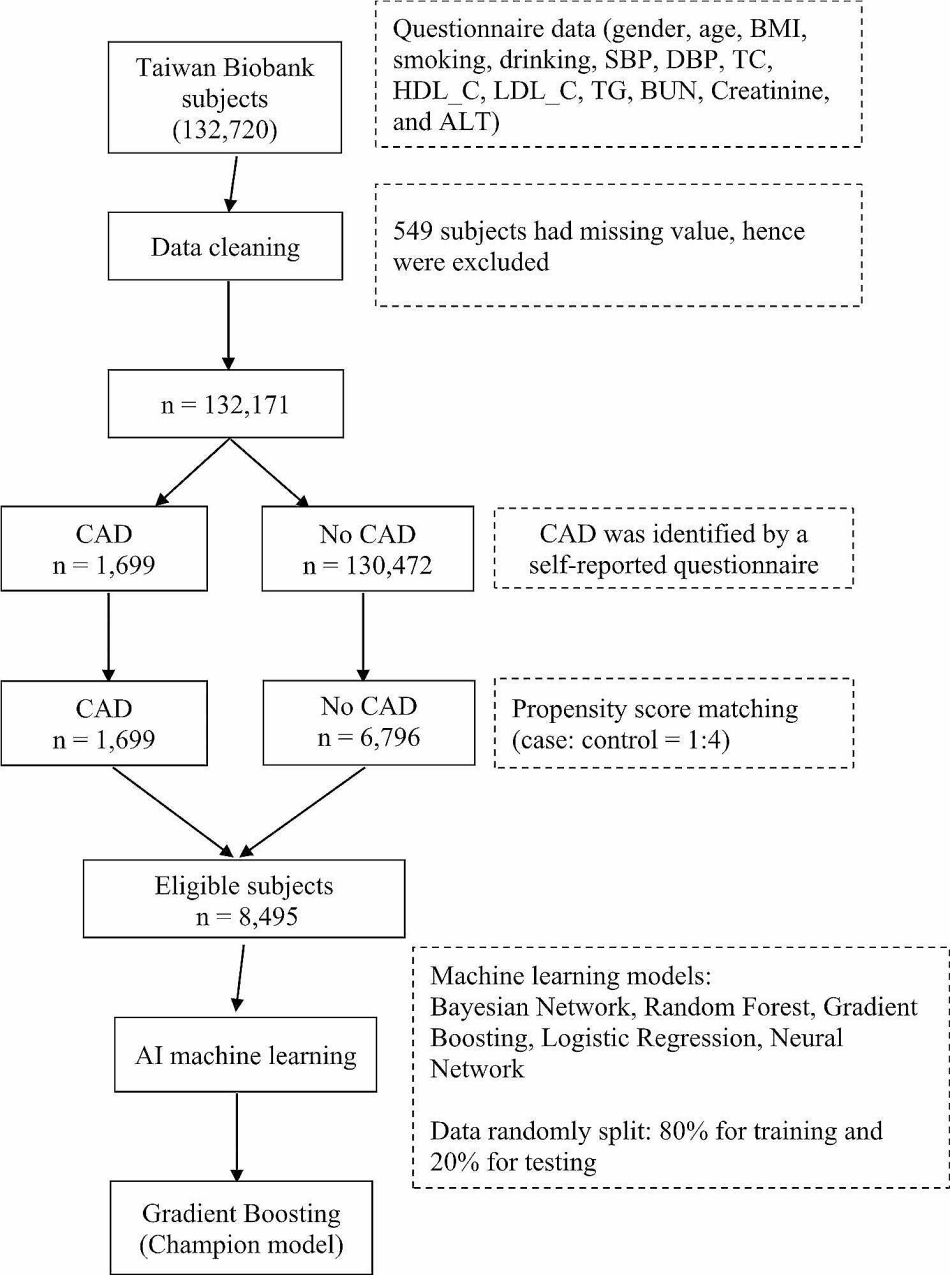
The pipeline describing the machine learning approach

The study utilized data from Taiwan Biobank, a large-scale database containing health-related information from Taiwanese adults. These individuals were assessed between 2008 and 2020. A total of 132,720 subjects were initially included in the dataset (Fig. 1). Subjects with missing values (*n* = 549) were excluded, resulting in a final study population of 132,171 subjects. The inclusion criteria focused on subjects with complete data across several variables. The primary outcome variable of interest was the presence of self-reported CAD among the study participants. A total of 1,699 subjects in the dataset reported a history of CAD. Approval for this study was provided by the institutional review board (IRB) of Chung Shan Medical University (CS1-20009). As the data were de-identified, informed consent was waived by the institutional review board.

The following features were included as predictors in the cardiovascular risk prediction models: body mass index (BMI), smoking status, gender, alcohol consumption (drinking), total cholesterol (T_CHO), high-density lipoprotein cholesterol (HDL_C), low-density Lipoprotein cholesterol (LDL_C), triglycerides (TG), blood urea nitrogen (BUN), creatinine, alanine aminotransferase (ALT), systolic blood pressure (SBP), diastolic blood pressure (DBP), and age [3]. Blood pressure measurements were obtained during assessment using an automated sphygmomanometer in a seated position. Two readings were taken and the average measurements were used for analysis. Individuals who had smoked consistently for at least six months and were currently smoking were classified as current smokers. Conversely, those who had never smoked or had quit smoking were categorized as nonsmokers. Similarly, individuals who habitually consumed more than 50 ml of alcohol per week for over six months were considered drinkers, whereas those with no alcohol intake, or who had abstained from drinking for more than six months, were considered nondrinkers. During assessment, blood pressure measurements were obtained using an automated sphygmomanometer in a seated position. Two readings were taken and the average measurements were used for analysis. Lipid panel measures were obtained using standardized enzymatic colorimetric assays.

### Propensity score matching

Propensity score matching was performed to balance potential confounders between subjects with and without CAD. A 1:4 matching ratio was applied (Fig. 1), resulting in a matched cohort of 8,495 subjects (1,699 with CAD and 6,796 without CAD) for subsequent analysis. This method facilitated the creation of a balanced dataset, enhancing the comparability between the CAD and no CAD groups and mitigating the influence of confounding variables. CAD status was determined based on self-reported questionnaires.

### Machine learning algorithms and data partitioning

A variety of machine learning-based algorithms were employed to construct cardiovascular risk prediction models using the aforementioned variables. These algorithms included: Bayesian Network, Logistic Regression, Random Forest, Neural Network, and Gradient Boosting. The dataset was partitioned into training (80%) and testing (20%) sets. The training set was used to train the machine learning models and the testing set was used to evaluate the performance of the models.

### Model training and evaluation

Each machine learning algorithm was trained on the training set using the selected predictors. Model performance was evaluated using metrics such as accuracy, sensitivity, specificity, area under the receiver operating characteristic curve (AUC-ROC), Youden’s index, and F1 score (a measure of the harmonic mean of precision and recall). The best-performing models were then evaluated on the independent testing set to assess their generalizability and predictive performance.

### Statistical analyses

We utilized SAS^®^ Viya^®^ (version 3.5, SAS Institute Inc., Cary, NC, USA) to automate the AI models. The dataset was split into training (80% of the data) and test (20% of the data) sets before developing machine learning models. Model performance was evaluated using the AUC metric, which assesses the ROC curve. We considered the various supervised learning models described above. An AUC value close to 1 indicated a well-performing model. CAD was assigned as the dependent variable. Continuous variables were presented as mean ± standard deviation, and categorical variables were expressed as frequencies and percentages. The importance of predictors in the Gradient Boosting model was determined based on their relative influence on the model’s predictive performance.

## Results

After excluding subjects with missing data, 1,699 individuals were identified with CAD through self-reported questionnaires, and propensity score matching yielded a final analysis set of 8,495 subjects (Table 1). The demographic and clinical features demonstrated significant distinctions between individuals with and without CAD. A larger proportion of those with CAD were men compared to women (66.69% vs. 33.31%, *p* < 0.001). Individuals with CAD were older on average compared to those without CAD (59.77 years vs. 49.58 years, *p* < 0.001). T_CHO, HDL_C, LDL_C, and TG were all significantly higher among individuals with CAD compared to those without CAD (*p* < 0.001 for all). A higher percentage of individuals with CAD were smokers and alcohol drinkers. Renal and liver function markers were also higher among individuals with CAD.

**Table 1:** Demographic features of the study population

	No CAD (*n* = 6,796)	CAD (*n* = 1,699)	*p*-value
**Gender**			< 0.001
Female	4411 (64.91)	566 (33.31)	
Male	2385 (35.09)	1133 (66.69)	
Age (y)	49.58 ± 10.90	59.77 ± 7.29	< 0.001
BMI (kg/m^2^)	24.20 ± 3.82	25.91 ± 3.67	< 0.001
Smoking (n, %)			< 0.001
No	5474 (80.55)	1007 (59.27)	
Yes	1322 (19.45)	692 (40.73)	
Drinking (n, %)			< 0.001
No	6185 (91.01)	1410 (82.99)	
Yes	611 (8.99)	289 (17.01)	
Blood pressure (mmHg)			
SBP	119.40 ± 17.94	129.80 ± 17.92	< 0.001
DBP	73.42 ± 11.02	76.35 ± 10.72	< 0.001
Cholesterol (mg/dL)			
T_CHO	195.50 ± 35.44	177.20 ± 38.33	< 0.001
HDL_C	55.00 ± 13.47	48.02 ± 11.76	< 0.001
LDL_C	120.70 ± 31.52	105.80 ± 32.76	< 0.001
TG	115.00 ± 91.36	139.10 ± 105.90	< 0.001
BUN (mg/dL)	13.15 ± 4.31	15.69 ± 5.99	< 0.001
Creatinine (mg/dL)	0.73 ± 0.41	0.91 ± 0.64	< 0.001
ALT (U/L)	24.04 ± 24.51	27.80 ± 20.29	< 0.001

Categorical variables: n (%). Continuous variables: Mean ± standard deviation. CAD: coronary artery disease, BMI: body mass index, SBP: systolic blood pressure, DBP: diastolic blood pressure, T_CHO: total cholesterol, HDL_C: high-density lipoprotein cholesterol, LDL_C: low-density lipoprotein cholesterol, TG: triglyceride, BUN: blood urea nitrogen, ALT: alanine aminotransferase

**Fig. 2:**
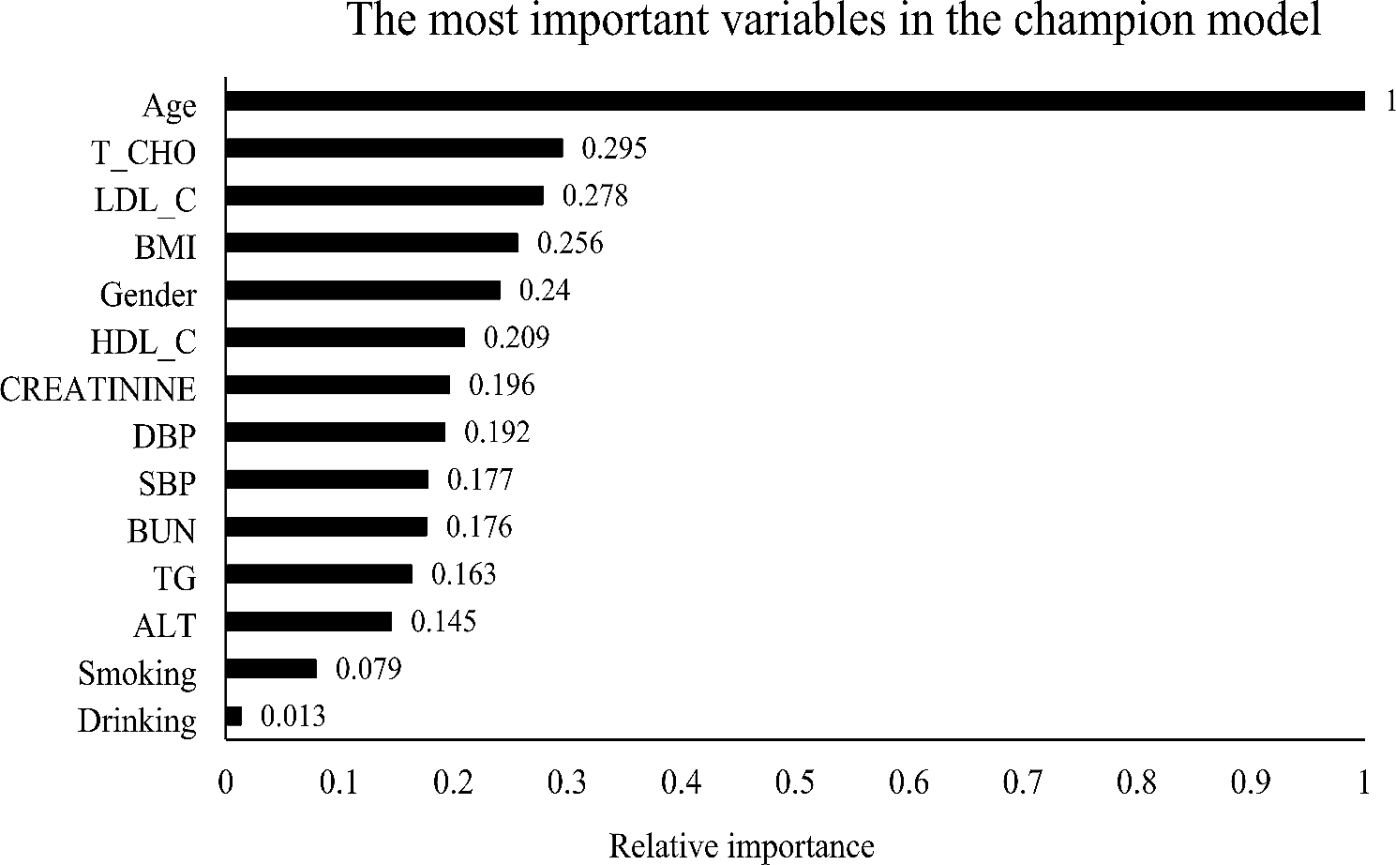
This plot shows the 14 most important variables, as determined by the Gradient Boosting (champion) model. The most important input for this model was age, followed by T_CHO.

The variable importance scores for the gradient-boosting champion model are displayed in Fig. 2. Among the 14 most influential features impacting the prediction of CAD, age emerged as the most relevant variable. This underscores the importance of age as a critical factor in the gradient-boosting model’s decision-making process, highlighting its relevance in CAD risk prediction within the studied dataset.

**Fig. 3:**
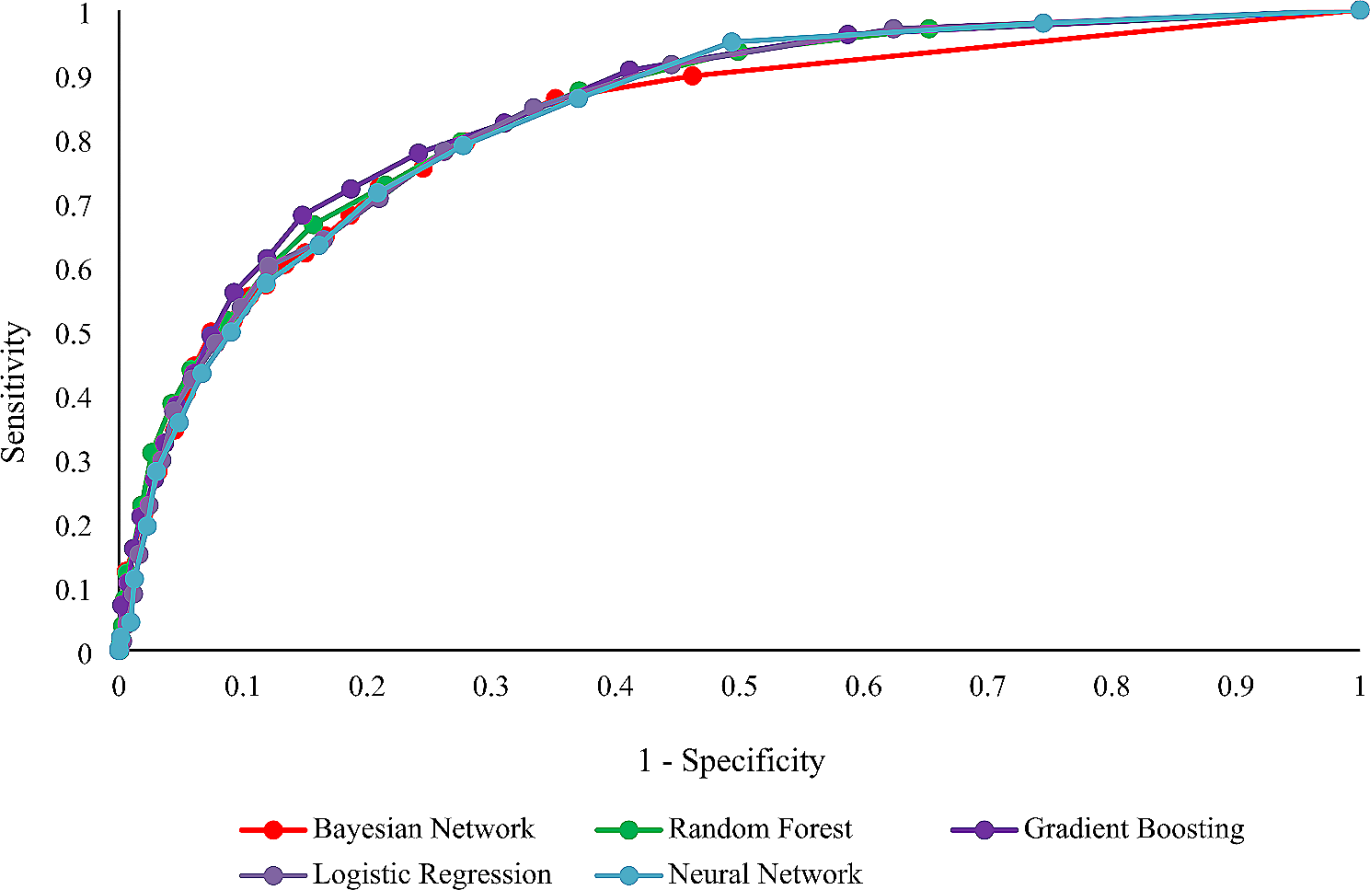
The AUROC for all models. (Gradient Boosting was the champion model)

Table 2 summarizes the performance metrics of various machine learning models in predicting CAD risk. The evaluation of predictive models indicated varied performances across different metrics. The Gradient Boosting model showcased the highest AUC value of 0.846, with a 95% CI of 0.819 to 0.873, suggesting it was the most effective in distinguishing between the classes. However, both the Bayesian Network and Random Forest models achieved the highest sensitivity, at 0.794 (95% CI: 0.751–0.837), indicating their precision in identifying true positives. Specificity was led by the Gradient Boosting model, reaching 0.759 (95% CI: 0.736–0.782), which denotes its strength in correctly identifying true negatives. This model also scored the highest in accuracy, with a value of 0.762 (95% CI: 0.742–0.782), and in F1 score, at 0.567 (95% CI: 0.543–0.591), reflecting its overall balanced performance in precision and recall. Logistic Regression and Neural Network models presented competitive performances with AUC values of 0.838 (95% CI: 0.811–0.865) and 0.836 (95% CI: 0.808–0.864), respectively. Although these models showed slightly lower sensitivity and specificity than the leading models, they remained robust in their predictive capabilities. The AUC-ROC curves for all models are shown in Fig. 3.

**Table 2:** Performance of predictive models under consideration

Algorithm	AUC (95% CI)	Sensitivity (95% CI)	Specificity (95% CI)	Accuracy (95% CI)	F1 (95% CI)
Bayesian Network	0.825 (0.797–0.853)	0.794 (0.751–0.837)	0.720 (0.696–0.744)	0.735 (0.714–0.756)	0.545 (0.521–0.569)
Random Forest	0.842 (0.815–0.869)	0.794 (0.751–0.837)	0.724 (0.700-0.748)	0.738 (0.717–0.759)	0.548 (0.524–0.572)
Gradient Boosting	0.846 (0.819–0.873)	0.776 (0.732–0.820)	0.759 (0.736–0.782)	0.762 (0.742–0.782)	0.567 (0.543–0.591)
Logistic Regression	0.838 (0.811–0.865)	0.779 (0.735–0.823)	0.738 (0.715–0.761)	0.746 (0.725–0.767)	0.552 (0.528–0.576)
Neural Network	0.836 (0.808–0.864)	0.788 (0.745–0.831)	0.723 (0.699–0.747)	0.736 (0.715–0.757)	0.544 (0.520–0.568)

Adjusted for gender, age, BMI, smoking, drinking, SBP, DBP, TC, HDL_C, LDL_C, TG, BUN, Creatinine, and ALT. The champion model was Gradient Boosting. The 95% confidence interval for the Sensitivity (0.756, 0.796), and the 95% confidence interval for the Specificity (0.749, 0.769

## Discussion

### Principal findings

The results of our study provide valuable insights into the demographic characteristics, risk factors, and predictive performance of machine learning models in assessing CAD risk in the studied population. Our analysis encompassed a range of performance metrics to evaluate the efficacy of different machine-learning algorithms. The gradient-boosting champion model emerged as the most effective in predicting CAD risk, achieving an AUC of 0.846. This high AUC value indicates the model’s strong discriminatory power in distinguishing between CAD-positive and CAD-negative cases. This is particularly notable as the value falls within the 0.8 to 0.9 range, considered accurate for predicting cardiovascular diseases with machine learning [5] In contrast, results from a previous study assessing atherosclerotic cardiovascular disease in Taiwan [22] showed that the eXtreme Gradient Boosting (XGBoost) and random forest models demonstrated the best performance with AUC-ROC values of 0.72 (0.68–0.76) and 0.73 (0.69–0.77) respectively, though not significantly better than other models.

Our study also showed solid results across other metrics, such as sensitivity, specificity, accuracy, and F1 Score, showcasing its reliability in CAD risk prediction, with AUC values ranging from 0.825 to 0.838. These models demonstrated varying degrees of sensitivity, specificity, accuracy, and F1 Score, indicating their differential capabilities in capturing CAD-related patterns and making accurate predictions. Based on prior research findings [23], future improvements in predicting recurrent cardiovascular disease risk may come from using comprehensive datasets and employing advanced, interpretable AI models, which could enhance precision and maintain clarity in decision-making processes. The adoption of AI models presents an opportunity to augment risk prediction capabilities. These strategic approaches signify potential pathways for advancing the precision and efficacy of cardiovascular event risk prediction in future research endeavors.

Our results further reveal that people with CAD often had higher risk factors such as age, BMI, high blood pressure, and poor lipid and renal function. Age was identified as a significant predictor of the disease. Our analysis also uncovered demographic differences in CAD prevalence, with men at higher risk, and lifestyle factors like smoking and drinking significantly affecting CAD risk. This underlines the need for lifestyle changes in CAD prevention strategies.

In Taiwan, the application of ML models for predicting CVD risk is gaining attention due to its potential to tailor preventive strategies and improve patient outcomes [24]. The country’s unique healthcare infrastructure, characterized by its National Health Insurance (NHI) system and TWB, offers extensive patient data, making it an ideal environment for testing these advanced predictive tools. In our study, we employed a neural network model with specific parameters designed to optimize performance while maintaining simplicity and interpretability. The architecture of the neural network consisted of a single hidden layer comprising 50 neurons. We utilized the hyperbolic tangent (Tanh) function as the activation function for the hidden layer due to its ability to introduce non-linearity and its effectiveness in handling a wide range of input values. The optimization of the network’s weights was performed using the Limited-memory Broyden-Fletcher-Goldfarb-Shanno (L-BFGS) algorithm, chosen for its efficiency in handling large-scale optimization problems and its suitability for neural network training. By explicitly detailing the neural network parameters, we aim to provide a clear framework that can be readily reproduced and built upon by future researchers. This transparency not only enhances the reproducibility of our findings but also facilitates a deeper understanding of the model’s behavior and performance characteristics.

Traditional algorithms for predicting cardiovascular disease have shown varying degrees of accuracy, with c statistics ranging from 0.65 to 0.85 [12, 13]. However, the integration of machine learning (ML) into healthcare for predicting CAD risk is showing promising results, with a notable increase in popularity due to its potential for more accurate predictions. A significant study utilizing data from the Multi-Ethnic Study of Atherosclerosis highlighted that ML algorithms outperformed both Cox proportional hazard models and traditional risk scores in CAD risk prediction [25]. Further research [23, 26] supports the advantage of ML in enhancing the accuracy of cardiovascular risk models through improved discrimination and calibration.

Previous explorations into CAD risk prediction have also ventured into the realm of genetic markers. One study introduced a combination of traditional risk factors, novel biomarkers, and a comprehensive set of genetic markers into ML models to predict coronary artery calcification [27]. Despite these efforts, the results yielded sensitivity and specificity rates of approximately 70% and 60%, respectively, suggesting that the addition of genetic data may not inherently boost prediction accuracy. The evidence suggests that ML algorithms may effectively harness traditional risk factors for CAD in the presence or absence of absence of new markers [23].

While much of the existing literature on machine learning in cardiovascular disease has focused on imaging-based approaches, routinely collected clinical biochemical indicators represent an important and underexplored area. A recent study has demonstrated the potential of machine learning models utilizing clinical data, such as blood biomarkers, to predict the presence and risk of cardiovascular diseases [28]. The authors developed a machine learning model based on 13 features, including lipid panel measures, to accurately identify individuals with coronary artery disease. Our findings add to this emerging body of research, highlighting the value of leveraging readily available clinical data for machine learning-based cardiovascular risk assessment. By constructing predictive models using common biochemical indicators, we can potentially provide a cost-effective and scalable approach to supporting clinical decision-making, complementing or even outperforming more resource-intensive imaging-based techniques in certain settings.

### Strengths and limitations

While our study points to the potential of machine learning in enhancing CAD risk prediction, we acknowledge its limitations, including its retrospective design and the need for further validation [21]. Furthermore, our investigation was hindered by a deficiency in data concerning disease severity within our study questionnaires. Consequently, we were unable to ascertain this crucial aspect. Finally, the CAD diagnosis was determined solely based on participants’ responses indicating they had ever been diagnosed with CAD by a doctor. We could not cross-reference this self-reported data with medical records or claims data from other data sources. The lack of objective clinical confirmation of the disease diagnosis might have introduced the potential for inaccuracies or biases. Participants may have under-reported or over-reported their CAD history, which could impact the reliability of our findings. Future research should aim to validate self-reported disease status against medical documentation to strengthen confidence in the results. Despite these shortcomings, our research underscores the value of machine learning, especially gradient boosting models, in providing accurate CAD risk assessments, which could improve clinical practices for early intervention and personalized care.

## Conclusions

In conclusion, these findings suggest that the Gradient Boosting model performed well in discriminating between CAD-positive and CAD-negative cases within a Taiwanese cohort, making it a promising tool for CAD risk prediction. Identifying key predictors supports the potential of targeted interventions and personalized medicine approaches in managing and preventing CAD.

## Data Availability

Taiwan Biobank data are available through **(**https://www.twbiobank.org.tw/*).* The data that support the findings of this study are available from the biobank but restrictions apply to the availability of these data, which were used under license for the current study, and so are not publicly available. Data are however available from the corresponding author (Yung-Po Liaw) upon reasonable request and with permission of Taiwan Biobank.

## Abbreviations

AI: artificial intelligence
CVD: cardiovascular disease
CRP: C-reactive protein
AUC: area under the curve
SNP: single nucleotide polymorphisms
BMI: body mass index
SBP: systolic blood pressure
DBP: diastolic blood pressure,serum creatinine
BUN: blood urea nitrogen
T_CHO: total cholesterol
TG: triglycerides
HDL_C: high-density lipoprotein-cholesterol
LDL_C: low-density lipoprotein-cholesterol
ALT: alanine aminotransferase
CAD: coronary artery disease

## References

[1] OECD, Organization WH. Health at a Glance: Asia/Pacific 20202020.

[2] Cupples L. Section 34: some risk factors related to the annual incidence of cardiovascular disease and death in pooled repeated biennial measurements. Framingham Heart Study: 30 Year Follow FollUp. 1987:1–22.

[3] Wilson PW, D’Agostino RB, Levy D, Belanger AM, Silbershatz H, Kannel WB. Prediction of coronary heart disease using risk factor categories. Circulation. 1998;97(18):1837–47. 9603539 10.1161/01.CIR.97.18.1837

[4] Damen JA, Pajouheshnia R, Heus P, Moons KG, Reitsma JB, Scholten RJ, et al. Performance of the Framingham risk models and pooled cohort equations for predicting 10-year risk of cardiovascular disease: a systematic review and meta-analysis. BMC Med. 2019;17:1–16. 31189462 10.1186/s12916-019-1340-7 PMC6563379

[5] Krittanawong C, Virk HUH, Bangalore S, Wang Z, Johnson KW, Pinotti R, et al. Machine learning prediction in cardiovascular diseases: a meta-analysis. Sci Rep. 2020;10(1):16057. 32994452 10.1038/s41598-020-72685-1 PMC7525515

[6] Conroy RM, Pyörälä K, Fitzgerald Ae, Sans S, Menotti A, De Backer G, et al. Estimation of ten-year risk of fatal cardiovascular disease in Europe: the SCORE project. Eur Heart J. 2003;24(11):987–1003. 12788299 10.1016/S0195-668X(03)00114-3

[7] Hippisley-Cox J, Coupland C, Vinogradova Y, Robson J, May M, Brindle P. Derivation and validation of QRISK, a new cardiovascular disease risk score for the United Kingdom: prospective open cohort study. BMJ. 2007;335(7611):136. 17615182 10.1136/bmj.39261.471806.55 PMC1925200

[8] Hippisley-Cox J, Coupland C, Vinogradova Y, Robson J, Minhas R, Sheikh A, et al. Predicting cardiovascular risk in England and Wales: prospective derivation and validation of QRISK2. BMJ. 2008;336(7659):1475–82. 18573856 10.1136/bmj.39609.449676.25 PMC2440904

[9] Ridker PM, Buring JE, Rifai N, Cook NR. Development and validation of improved algorithms for the assessment of global cardiovascular risk in women: the Reynolds risk score. JAMA. 2007;297(6):611–9. 17299196 10.1001/jama.297.6.611

[10] Woodward M, Brindle P, Tunstall-Pedoe H. Adding social deprivation and family history to cardiovascular risk assessment: the ASSIGN score from the Scottish Heart Health Extended Cohort (SHHEC). Heart. 2007;93(2):172–6. 17090561 10.1136/hrt.2006.108167 PMC1861393

[11] D’Agostino Sr RB, Vasan RS, Pencina MJ, Wolf PA, Cobain M, Massaro JM, et al. General cardiovascular risk profile for use in primary care: the Framingham Heart Study. Circulation. 2008;117(6):743–53. 18212285 10.1161/CIRCULATIONAHA.107.699579

[12] Lloyd-Jones DM. Cardiovascular risk prediction: basic concepts, current status, and future directions. Circulation. 2010;121(15):1768–77. 20404268 10.1161/CIRCULATIONAHA.109.849166

[13] Pennells L, Kaptoge S, Wood A, Sweeting M, Zhao X, White I, et al. Equalization of four cardiovascular risk algorithms after systematic recalibration: individual-participant meta-analysis of 86 prospective studies. Eur Heart J. 2019;40(7):621–31. 30476079 10.1093/eurheartj/ehy653 PMC6374687

[14] D’Agostino RB, Grundy S, Sullivan LM, Wilson P, Group CRP. Validation of the Framingham coronary heart disease prediction scores: results of a multiple ethnic groups investigation. JAMA. 2001;286(2):180–7. 11448281 10.1001/jama.286.2.180

[15] Thomsen TF, McGee D, Davidsen M, Jørgensen T. A cross-validation of risk-scores for coronary heart disease mortality based on data from the Glostrup Population studies and Framingham Heart Study. Int J Epidemiol. 2002;31(4):817–22. 12177028 10.1093/ije/31.4.817

[16] Hense H-W, Schulte H, Löwel H, Assmann G, Keil U. Framingham risk function overestimates risk of coronary heart disease in men and women from Germany—results from the MONICA Augsburg and the PROCAM cohorts. Eur Heart J. 2003;24(10):937–45. 12714025 10.1016/S0195-668X(03)00081-2

[17] Liu J, Hong Y, D’Agostino Sr RB, Wu Z, Wang W, Sun J, et al. Predictive value for the Chinese population of the Framingham CHD risk assessment tool compared with the Chinese Multi-provincial Cohort Study. JAMA. 2004;291(21):2591–9. 15173150 10.1001/jama.291.21.2591

[18] Okwuosa TM, Mallikethi-Reddy S, Jones DML. Strategies for treating lipids for prevention: risk stratification models with and without imaging. Best Pract Res Clin Endocrinol Metab. 2014;28(3):295–307. 24840260 10.1016/j.beem.2014.01.004

[19] Beam AL, Kohane IS. Big data and machine learning in health care. JAMA. 2018;319(13):1317–8. 29532063 10.1001/jama.2017.18391

[20] Malik P, Pathania M, Rathaur VK. Overview of artificial intelligence in medicine. J Family Med Prim care. 2019;8(7):2328–31. 31463251 10.4103/jfmpc.jfmpc_440_19 PMC6691444

[21] Banerjee A, Chen S, Fatemifar G, Zeina M, Lumbers RT, Mielke J, et al. Machine learning for subtype definition and risk prediction in heart failure, acute coronary syndromes and atrial fibrillation: systematic review of validity and clinical utility. BMC Med. 2021;19(1):1–14. 33820530 10.1186/s12916-021-01940-7 PMC8022365

[22] Hsiao YC, Kuo CY, Lin FJ, Wu YW, Lin TH, Yeh HI, et al. Machine learning models for ASCVD Risk Prediction in an Asian Population - How to validate the Model is important. Acta Cardiol Sin. 2023;39(6):901–12. 38022427 10.6515/ACS.202311_39(6).20230528A PMC10646597

[23] Westerlund AM, Hawe JS, Heinig M, Schunkert H. Risk Prediction of Cardiovascular events by exploration of Molecular Data with Explainable Artificial Intelligence. Int J Mol Sci. 2021;22(19):10291. 34638627 10.3390/ijms221910291 PMC8508897

[24] Lin YC, Tsai CH, Hsu HT, Lin CH, editors. Using Machine Learning to Analyze and Predict the Relations Between Cardiovascular Disease Incidence, Extreme Temperature and Air Pollution. 2021 IEEE 3rd Eurasia Conference on Biomedical Engineering, Healthcare and Sustainability (ECBIOS); 2021 28–30 May 2021.

[25] Ambale-Venkatesh B, Yang X, Wu CO, Liu K, Hundley WG, McClelland R, et al. Cardiovascular event prediction by machine learning: the multi-ethnic study of atherosclerosis. Circul Res. 2017;121(9):1092–101. 10.1161/CIRCRESAHA.117.311312 PMC5640485 28794054

[26] Cho S-Y, Kim S-H, Kang S-H, Lee KJ, Choi D, Kang S, et al. Pre-existing and machine learning-based models for cardiovascular risk prediction. Sci Rep. 2021;11(1):1–10. 33903629 10.1038/s41598-021-88257-w PMC8076166

[27] Sun YV, Bielak LF, Peyser PA, Turner ST, Sheedy PF, Boerwinkle E, et al. Application of machine learning algorithms to predict coronary artery calcification with a sibship-based design. Genetic Epidemiology: Official Publication Int Genetic Epidemiol Soc. 2008;32(4):350–60. 10.1002/gepi.20309 PMC2828904 18271057

[28] Weng S, Chen J, Ding C, Hu D, Liu W, Yang Y et al. Utilizing machine learning algorithms for the prediction of carotid artery plaques in a Chinese population. Front Physiol. 2023;14. 10.3389/fphys.2023.1295371 PMC10657816 38028761

